# Copeptin in hyponatremia: is there a role for this biomarker in the diagnostic workup?

**DOI:** 10.1007/s12020-018-1557-9

**Published:** 2018-03-01

**Authors:** Marco Baldrighi, Luigi Mario Castello, Ettore Bartoli

**Affiliations:** Department of Translational Medicine, Università del Piemonte Orientale, Novara, Italy

Hyponatremia (plasma sodium concentration below 136 mEq/L) is the most common electrolyte derangement among both hospitalized [1] and emergency department (ED) patients [2], and its treatment represents a major issue both for internal medicine and emergency physicians. However, since different forms of hyponatremia demand different therapeutic approaches, a proper treatment is always the result of a correct diagnosis [3].

Volume status assessment is the first crucial step in the diagnostic and therapeutic algorithm of hyponatremia. Although history, clinical signs, and routine laboratory tests remain the most widely used diagnostic tools, they have shown poor accuracy in the definition of volume status [4]. The need for more reliable tools to assess patients’ volume-fueled, the research on biomarkers of hyponatremia with the specific intent to help the physician in the first steps of diagnosis, which usually take place in the ED. Among these, copeptin has been the most thoroughly studied.

Copeptin is a 39 amino acids glycoprotein that contains the C-terminal region of pre-pro-vasopressin. As a stabile product of the cleavage of AVP precursor, copeptin is released in equimolar amounts with the hormone [5] and can thus be a surrogate marker of AVP secretion in clinical practice [6]. The plasma copeptin concentration varies with plasma osmolality and is affected by many different diseases, such as acute coronary syndrome, stroke, shock, and trauma. *Endocrine* has recently published various papers concerning the diagnostic role of copeptin in both posterior [7] and anterior pituitary disfunctions [8, 9].

In spite of the promising results of recent studies focused on the diagnostic and prognostic role of copeptin in the setting of acute dyspnea-related both to heart failure [10–12] and acute exacerbations of chronic obstructive pulmonary disease [10, 11, 13]; the findings in the field of hyponatremia are more controversial.

In 2009 Fenske et al. demonstrated through a prospective study a very high variability in copeptin levels of 106 hyponatremic patients, but suggested that the ratio between plasma copeptin and urinary sodium (_P_(copeptin)-to-_U_(Na^+^) ratio), could help distinguishing SIADH-related hyponatremia from that following either real or virtual volume depletion (hypovolemic and hypervolemic, respectively) [14]. In 2011 Nigro et al. performed a secondary analysis of three previous prospective studies and found that copeptin levels were lower in hypovolemic hyponatremia and progressively raised in euvolemic and hypervolemic patients [15].

In 2017 the same group published the results of a large prospective multicentre study involving 298 patients with severe hyponatremia (plasma sodium <125 mEq/L). Actually, copeptin levels were significantly different among the various causes of hyponatremia, but the post-hoc comparisons showed that only hyponatremia due to primary polydipsia could be effectively distinguished from hyponatremia due to other causes on the basis of copeptin cut-off. Moreover, in contrast to the results of their previous work, hypovolemic patients were found to have the highest copeptin levels. Interestingly, clinical volume status assessment showed a better performance in predicting hypovolemic hyponatremia than the biomarker [16].

Nigro et al. concluded that the diagnostic utility of copeptin is limited [16]. Our research group essentially agrees with this final statement, particularly if copeptin levels are obtained in an emergency medicine context. In fact, in the last few years we carried out many studies focused on the diagnosis and treatment of hyponatremia in the ED [17, 18]. For example, data we obtained from 22 patients with hypovolemic hyponatremia confirmed the wide variability of copeptin levels even in the same subgroup of patients (IQR 9.71–100.95 pmol/L) [18]. In the same study, we also found that only 59.1% of our hypovolemic patients had a _P_(copeptin)-to-_U_(Na^+^) ratio reported to be consistent with hypovolemia according to the cut-off value proposed by Fenske et al. [14].

Unpublished data obtained from a secondary analysis of 18 euvolemic and hypervolemic patients with hyponatremia enrolled in another ED trial [17], once again demonstrates a wide variability in copeptin levels without significant differences between euvolemic and hypervolemic patients.

In our studies, patients were divided into larger and more heterogeneous groups than those created by Nigro et al. according to a precise, definitive aetiologic diagnosis put on the basis of many elements comprehensive of the response to treatment [16]. However, we think that this better reflects what usually happens in an ED, where a causative diagnosis is mostly presumptive and patients can only be categorized according to their volume status. On the other hand, in more selected groups of patients, copeptin has shown a better diagnostic potential (for example to detect paraneoplastic SIADH in cancer patients [7]).

Concluding, we think that copeptin cannot be a useful biomarker in the initial approach to hyponatremia in the ED. The main explanation could be that copeptin levels increase in response to most of the acute as well as to stress conditions. As these both are present almost unerringly in ED patients, the regulation of AVP secretion by osmotic and non-osmotic stimuli (i.e., hypertonicity and volume depletion, respectively) can be disrupted, thus undermining the pathophysiologic rationale that underlies the use of copeptin as a biomarker in hyponatremia.
